# Artificial intelligence for surgical data management and decision support: lessons from wound care

**DOI:** 10.3389/frai.2025.1718436

**Published:** 2025-11-13

**Authors:** Esteban Zavaleta-Monestel, Sebastián Arguedas-Chacón, Katherine Cordero-Bermúdez

**Affiliations:** 1 Health Research Department, Hospital Clínica Bíblica, San José, Costa Rica; 2 Department of General Surgery, Rafael Ángel Calderón Guardia Hospital, Costa Rican Social Security Fund (CCSS), San José, Costa Rica; 3 Hernia and Abdominal Wall Rehabilitation Clinic, Clínica Bíblica Hospital, San José, Costa Rica

**Keywords:** natural language processing, artificial intelligence, clinical decision-making, wound healing, outcome assessment, health care

## Introduction

The surge in surgical data has made converting raw documentation, operative notes, pathology reports, laboratory results, and outcomes registries into actionable clinical insights a considerable challenge for clinical care and research. Though these sources hold valuable information, manual chart review is slow, inconsistent, and unsustainable in high-volume environments (Murff et al., 2011; Mellia et al., 2021). Surgeons now dedicate a significant portion of their workday to documentation instead of direct patient care. A time-and-motion study observed that nearly half of physicians' working hours are consumed by electronic health record (EHR) tasks (Sinsky et al., 2016). These findings underscore the need for innovative solutions that enhance documentation without compromising care quality (Joukes et al., 2018).

Intelligent data-processing methods are beginning to bridge this gap. For example, natural language processing (NLP), which allows computers to interpret and generate human language, has been used in medicine for over a decade. Early research showed stronger detection of postoperative complications compared to billing-code reviews (Murff et al., 2011). More recently, large language models (LLMs), such as ChatGPT, have demonstrated the ability to rapidly and accurately analyze narrative clinical text (Chen et al., 2022; Dagli et al., 2024; Friedman et al., 2025).

AI applications go beyond text interpretation. Similarly, advances in image recognition, intraoperative navigation, and robotic assistance are evolving, positioning structured data management as pivotal in surgery's digital transformation (Beyaz et al., 2025). Collectively, these breakthroughs foreshadow a future where automated information extraction aids perioperative decision-making and redesigns surgical workflows (Han et al., 2025).

## Evidence from chart review

For over a decade, NLP has proven valuable in surgical research. Automated text analysis has outperformed billing-code reviews in detecting postoperative complications, achieving higher sensitivity without loss of specificity (Murff et al., 2011). A meta-analysis confirmed that NLP detects complications more accurately than manual methods while maintaining comparable specificity (Mellia et al., 2021).

Recent work has evolved from feasibility to refinement. For example, a data-extraction pipeline developed for breast cancer reports identified 48 outcome variables with near-human accuracy, achieving *F*-scores above 0.90 for most measures. F-score is a statistic that combines sensitivity and precision to measure accuracy (Chen et al., 2022). In another instance, a framework that combined NLP and LLM integration for spinal surgery attained near-perfect sensitivity for key operative variables, reduced review time by more than 3,000-fold, and dramatically lowered costs (Dagli et al., 2024).

Taken together, these studies indicate that intelligent data-processing tools are evolving from experimental demonstrations to realistic clinical applications. However, routine implementation across hospitals remains uncommon and will require further validation in real-world settings.

## Wound care as a case study

Plastic surgery and wound care in particular present unique documentation challenges due to their reliance on narrative detail. In a recent study, employing an LLM reduced the average chart review time from 7.56 to 1.03 min per case, all while maintaining an overall accuracy of 95.7% (ranging from 74.7% to 98.6% across variables). Furthermore, the model generated wound summaries that closely echoed clinician notes (Friedman et al., 2025).

Furthermore, these findings are consistent with results from other surgical areas, including breast cancer and spine surgery, indicating that wound care exemplifies a broader trend toward data-driven surgical documentation (Chen et al., 2022; Dagli et al., 2024). Wound care is particularly illustrative because it integrates diverse data types, involves multidisciplinary teams, and addresses both functional and aesthetic outcomes.

This complexity matches challenges in other plastic surgery subspecialties, such as breast reconstruction. Here, clinical data are unstructured, and outcomes include quality of life, cosmetic satisfaction, and survival (Spoer et al., 2022; Rugină et al., 2025). Recent reviews show wound care is an ideal area for machine learning and language-based systems. Applications range from wound assessment and prognosis to treatment personalization and outcome prediction (Ganesan et al., 2024).

As a result, wound care offers a practical model for evaluating the implementation of intelligent data tools prior to broader adoption. Automation in this context can improve efficiency without compromising accuracy, even in domains characterized by free-text narratives (Friedman et al., 2025).

## Toward decision support

Improving efficiency is only the first step; the real promise of intelligent systems lies in their ability to support informed decision-making in surgery. Beyond documentation, several experimental tools are now being tested for intraoperative use, including platforms that interpret real-time imaging and assist robotic procedures (Byrd IV and Tignanelli, 2024; Matheny et al., 2020). These applications remain in early development and require validation and regulatory review before being used in routine clinical settings (Liang et al., 2025).

For instance, one NLP-based system accurately predicted unplanned intensive care admissions in elective neurosurgical patients by analyzing free-text clinical notes. Another machine learning approach, applied to ventral hernia repairs, identified recurrence, surgical site occurrence, and 30-day readmission rates using preoperative EMR data (Hassan et al., 2022).

Together, these advances indicate a transition from retrospective data review to real-time risk assessment. This shift demonstrates that digital and learning-based systems can evolve from record-keeping to clinical decision support, enabling surgeons to anticipate complications rather than only documenting them (Ive et al., 2025).

## Challenges and governance

Despite these advances, substantial limitations persist. Most current studies are retrospective, single-center, and focus on feasibility; thus, their relevance to other institutions using diverse EHR systems and documentation styles is constrained. Moreover, model performance fluctuates across different data types, and occasional errors, such as hallucinations or misclassifications, underscore the continued necessity for human supervision (Mellia et al., 2021; Liang et al., 2025).

Bias and equity also present significant concerns. Numerous algorithms are built on data from high-income countries and may not represent patient populations in low- and middle-income regions. Deploying these systems globally without adaptation and tailoring increases the risk of reinforcing health disparities. Additionally, differences in wound healing, influenced by comorbidities, nutrition, or access to follow-up care, highlight the importance of population-specific validation (Ganesan et al., 2024).

Robust governance frameworks are crucial. Intelligent data systems must offer transparency, undergo validation across institutions, and adhere to international standards. Privacy and data security are vital. Clinicians may be reluctant to adopt platforms that rely on external processing, emphasizing the need for secure, locally hosted solutions (Liang et al., 2025; European Parliament and Council, 2024).

Regulators are starting to act. In the European Union, the AI Act establishes risk-based requirements for medical software, including transparency, documentation, and post-market monitoring (European Parliament and Council, 2024). In the United States, the Food and Drug Administration (FDA) has issued guidance for adaptive software that uses machine learning. This signals that surgical data tools will face more regulatory scrutiny (US Food Drug Administration, 2025).

Finally, patient trust remains central. A recent mixed-methods study found that while most patients view AI-assisted decision support positively, concerns persist about safety, accountability, and clinician oversight. Unless these issues are directly addressed, clinical adoption is likely to remain cautious and uneven (Ben Hmido et al., 2025).

## Discussion

Current evidence shows that intelligent data systems can strengthen surgical data management by improving both efficiency and accuracy. Systematic reviews and meta-analyses confirm that NLP consistently outperforms traditional chart review in identifying perioperative outcomes (Mellia et al., 2021). More recent studies demonstrate that advanced NLP pipelines and LLM-based frameworks can reliably extract complex operative and pathology data across diverse specialties, including oncology and spine surgery (Chen et al., 2022; Dagli et al., 2024). These results indicate that digital tools in surgery are supported not only by conceptual promise but also by practical demonstrations of value.

Wound care exemplifies this progress clearly. Friedman et al. found that using a language model reduced chart review time by more than 80% while maintaining high accuracy, even in documentation that relies heavily on narrative text (Friedman et al., 2025). When viewed alongside findings from breast cancer and spine surgery, this evidence highlights how automated text analysis can transform unstructured information into organized data that supports both patient care and research (Chen et al., 2022; Dagli et al., 2024).

There are still significant challenges. Most studies remain retrospective, single-institution, and rarely explore the realities of real-time workflow integration. Occasional errors, such as hallucination or misclassification, require human oversight and external validation. The lack of standardized reporting frameworks also limits comparison across studies and slows clinical translation (Liang et al., 2025). Ethical, privacy, and governance issues are equally important. Without transparent validation, clear accountability, and equitable implementation, digital systems risk eroding rather than building trust among clinicians and patients (European Parliament and Council, 2024; US Food Drug Administration, 2025; Ben Hmido et al., 2025).

Future progress will depend not only on validation but also on robust infrastructure. Multi-institutional collaborations can generate diverse datasets that capture variations in surgical practice, while standardized benchmarks can help clinicians compare model performance across institutions (Li et al., 2025). Like clinical trial registries, shared databases for algorithm performance could promote transparency, fairness, and reproducibility in real-world applications.

International coordination will also be essential. Without harmonized regulatory frameworks, global deployment of surgical data systems could become fragmented, slowing innovation and disadvantaging regions with fewer resources (Mennella et al., 2024; Rosenthal et al., 2025).

**Figure 1:**
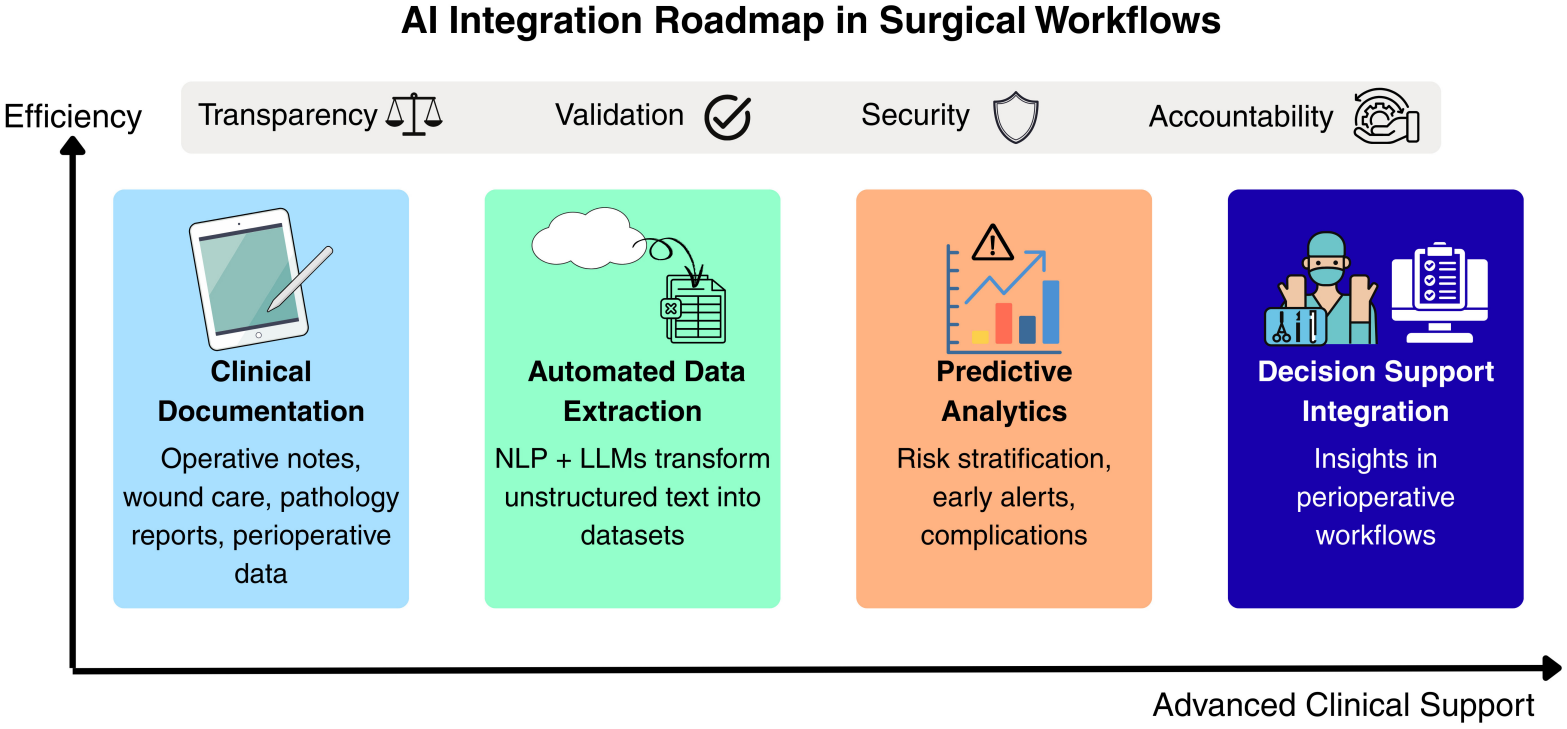
Conceptual roadmap for integrating intelligent data systems into surgical workflows.

Figure 1 summarizes this staged trajectory, showing a roadmap for integrating intelligent tools into surgical workflows, from initial documentation support toward advanced perioperative decision assistance guided by transparency, validation, security, and accountability.

Looking ahead, collaboration between institutions and clear performance benchmarks will be key to ensuring safe adoption. The evolution of these systems should follow a structured path: beginning with documentation efficiency, advancing to predictive analytics for complication detection, and eventually integrating into perioperative decision support. Achieving this will require multidisciplinary cooperation, regulatory guidance, and a firm commitment to preserving clinical judgment while embracing the benefits of automation. The surgical community must take an active role in defining how these technologies are validated and applied so that innovation translates into safer and more efficient patient care (Maleki Varnosfaderani and Forouzanfar, 2024).

## Conclusions

Intelligent data systems emerge as practical tools in surgery, improving both efficiency and accuracy in data extraction across multiple specialties. However, most current studies remain retrospective and single-center, highlighting the need for broader validation, standardized benchmarks, and strong governance. A gradual pathway, from documentation support to risk prediction and perioperative decision assistance, will be key to responsible implementation. With collaboration across disciplines and active regulatory guidance, these technologies can help transform innovation into safer and more efficient surgical care.
